# Molecular analysis of metallo-beta-lactamase-producing *Pseudomonas aeruginosa* in Switzerland 2022–2023

**DOI:** 10.1007/s10096-024-04752-8

**Published:** 2024-01-18

**Authors:** Jacqueline Findlay, Otavio Hallal Ferreira Raro, Laurent Poirel, Patrice Nordmann, NARA Network, R. Lienhard, L. Vonallmen, C. Schilt, A. Scherler, K. Lucke, M. Jutzi, M. Reichmuth, U. Schibli, C. Fricker, S. Pranghofer, G. Greub, D. Blanc, A. Vitale, B. Lemaire, M. Fatoux, M. Tritten, L. Rumebe, N. Liassine, G. Jost, N. Wohlwend, D. Schultze, K. Burren, A. Westers, M. Imperiali, L. Pozzi, D. Balzari, G. Vaninetti, C. Cirillo, V. Gaia, E. Pianezzi, G. Martinetti Lucchini, F. Baggi Menozzi, A. Jayol, C. Guyon, D. Hyden, M. Maitrejean, V. Deggi-Messmer, D. Bandeira, C. Fournier, S. Pfister, C. Nusbaumer, L. Bertaiola Monnerat, J. Schrenzel, G. Renzi, A. Cherkaoui, D. Andrey, S. Emonet, M. Eyer, R. Maret, A. Belo, D. Mabillard, M. Moraz, K. Herzog, V. Gisler, E. Hitz, M. Oberle, C. Castelberg, H. Fankhauser, S. Graf, N. Dubey, C. Guler, M. Schoenenberger, U. Karrer, F. Piran, C. Andreutti, M. Dessauges, T. Schmid, B. Suterbuser, I. Mitrovic, E. Gruner, V. Bruderer, P. Staehli, B. Schnell, C. O. Marti, I. Steffen, A. Imhof, B. Preiswerk, V. Dilorenzo, C. Payen, D. Boschung, L. Comte, M. Schacher, M. Brandenberger, C. Zowa, C. Zehnder, B. Mathis, L. Basilico, G. Togni, P. Minkova, Y. Born, M. Kuegler, V. Povolo, S. Droz, M. Elzi, C. Casanova, D. Goldenberger, P. Keller, C. Lang, A. Blaich, S. Schmid, B. Ivan, A. Egli, S. Mancini, O. Dubois, K. Narr, S. Schoch, S. Ellenberger, S. Seiffert

**Affiliations:** 1 Medical and Molecular Microbiology, Faculty of Science and Medicine, University of Fribourg, Chemin du Musée 18, CH-1700 Fribourg, Switzerland; 2 Swiss National Reference Center for Emerging Antibiotic Resistance (NARA) Network, Swiss National Reference Center for Emerging Antibiotic Resistance (NARA), University of Fribourg, Fribourg, Switzerland

**Keywords:** *Pseudomonas aeruginosa*, Metallo-beta-lactamase, Carbapenems, Epidemiology

## Abstract

**Objectives:**

The occurrence of metallo-beta-lactamase-producing *Pseudomonas aeruginosa* (MBL-PA) isolates is increasing globally, including in Switzerland. The aim of this study was to characterise, phenotypically and genotypically, the MBL-PA isolates submitted to the Swiss National Reference Center for Emerging Antibiotic Resistance (NARA) reference laboratory over a 12-month period from July 2022 to July 2023.

**Methods:**

Thirty-nine non-duplicate MBL-PA Isolates were submitted to NARA over the study period from across Switzerland. Susceptibility was determined by broth microdilution according to EUCAST methodology. Whole-genome sequencing was performed on 34 isolates. Sequence types (STs) and resistance genes were ascertained using the Centre for Genomic Epidemiology platform. MBL genes, *bla*_NDM-1_, *bla*_IMP-1_, and *bla*_VIM-2_, were cloned into vector pUCP24 and transformed into *P. aeruginosa* PA14.

**Results:**

The most prevalent MBL types identified in this study were VIM (21/39; 53.8%) followed by NDM (11/39; 28.2%), IMP (6/39; 15.4%), and a single isolate produced both VIM and NDM enzymes. WGS identified 13 different STs types among the 39 isolates. They all exhibited resistance to cephalosporins, carbapenems, and the beta-lactam-beta-lactamase inhibitor combinations, ceftolozane-tazobactam, ceftazidime-avibactam, imipenem-relebactam, and meropenem-vaborbactam, and 8 isolates were cefiderocol (FDC) resistant. Recombinant *P. aeruginosa* strains producing *bla*_NDM-1_, *bla*_IMP-1_, and *bla*_VIM-2_ exhibited FDC MICs of 16, 8, and 1 mg/L, respectively.

**Conclusions:**

This study showed that the MBL-PA in Switzerland could be attributed to the wide dissemination of high-risk clones that accounted for most isolates in this study. Although FDC resistance was only found in 8 isolates, MBL carriage was shown to be a major contributor to this phenotype.

**Supplementary Information:**

The online version contains supplementary material available at 10.1007/s10096-024-04752-8.

## Introduction

*Pseudomonas aeruginosa* is a major cause of nosocomial infections, particularly in immune-compromised patients, and is associated with considerable morbidity and mortality [1, 2]. The increasing global incidence of carbapenem-resistant *P. aeruginosa* (CRPA) is a cause of great concern since infections caused by such multidrug-resistant organisms often leave very few viable therapeutic options [1–3]. Carbapenem resistance in *P. aeruginosa* can attributed to a number of mechanisms including permeability defects, the production of carbapenemases, and the over-expression of genes encoding efflux pumps [4, 5]. Numerous epidemiological studies have shown that CRPA infections are predominantly related to high-risk clones producing carbapenemases along with other beta-lactamase genes, in addition to possessing other non-ß-lactamase-related resistance mechanisms [3]. ST235 is globally the most prevalent high-risk clone and has been associated with various resistance mechanisms including the production of diverse carbapenemase types, but predominantly metallo-beta-lactamases (MBLs) [6]. VIM-, NDM-, and IMP-type MBLs are the most common MBL types in CRPA, and infections caused by MBL-producing *P. aeruginosa* (MBL-PA) are particularly challenging since these enzymes confer resistance to all beta-lactams, including all currently available beta-lactam-beta-lactamase inhibitor (BLBLI) combinations, with the exception of aztreonam (ATM), and the recently approved siderophore antibiotic, cefiderocol (FDC) [7]. However, MBL-PA are often found to be resistant to ATM due to the production of other beta-lactamases (e.g. ESBLs, and particularly GES-type enzymes) and/or overexpression of the intrinsic *bla*_PDC_ gene, thereby limiting the use of this antimicrobial [8]. Additionally, resistance to FDC has been reported to be associated with the carriage of NDM-type enzymes and/or mutations in iron transporter systems in some clinical isolates [9, 10]. To contend with so few available therapeutic options, several novel BLBLI combinations are currently under development for the treatment of MBL-PA including aztreonam-avibactam (ATM-AVI), cefepime-taniborbactam (FEP-TAN), and cefepime-zidebactam (FEP-ZID)—all of which are currently in phase 3 clinical trials. ATM-AVI combines ATM with AVI, a diazabicyclooctane (DBO), which allows the restoration of susceptibility in MBL-producers that also produce other class A, C, and some class D beta-lactamases [11]. FEP-TAN, combining a 4th-generation cephalosporin with a bicyclic boronate, has been shown to exhibit excellent activity against Ambler class A, C, and D beta-lactamases as well as the class B beta-lactamases, NDM and VIM, but notably has no activity against IMP enzymes [12]. The FEP-ZID combination similarly includes FEP but with ZID, a bicyclo-acyl hydrazide compound, which exhibits dual activity by inhibiting the hydrolytic activity of many beta-lactamases (classes A, C, and D) and additionally possessing significant antimicrobial activity on its own by binding PBP-2—subsequently rendering this BLBLI to be effective against MBL-PA [13]. These new BLBLIs, if approved, will offer a much needed expansion of the limited armamentarium against MBL-PA, and subsequently, any arising resistance will need to be closely monitored.

The aims of our study were (i) to characterise, both phenotypically and genotypically, all of the MBL-producing *P. aeruginosa* isolates submitted to the Swiss National Reference Center for Emerging Antibiotic Resistance (NARA) reference laboratory over a 12-month period from July 2022 to July 2023 for their resistance traits and (ii) to determine their respective susceptibility to all currently available and recently developed therapeutical options, and also to some of the new BLBLIs.

## Materials and methods

### Bacterial isolates, identification, and susceptibility testing

Isolates exhibiting resistance to carbapenems were submitted to the NARA reference laboratory from hospitals and clinics throughout Switzerland, over a 12-month period, from July 2022 to July 2023. Patient and isolation source data was obtained from the accompanying request forms sent by referring laboratories. Species identification was confirmed using API-20NE tests (bioMérieux, https://www.biomerieux.com). Susceptibility testing was performed by broth microdilution, and results were interpreted in accordance with EUCAST guidelines [14]. To investigate the contribution of efflux mechanisms to FEP-TAN and FEP-ZID resistance, MICs were also performed in the presence of 25 mg/L phenylalanine-arginine β-naphthylamide (PAβN). Carbapenemase activity was detected by Carba NP test [15], and carbapenemase gene alleles were confirmed by PCR and subsequent Sanger sequencing.

### Cloning experiments

MBL genes, *bla*_NDM-1_, *bla*_IMP-1_, and *bla*_VIM-2_, were amplified and cloned into high copy number vector pUCP24 [16], using primers listed in Table S1, before transformation into *P. aeruginosa* PA14.

### Whole-genome sequencing and analyses

Whole-genome sequencing (WGS) was performed on a subset of 34, randomly selected, isolates on a MiSeq instrument (Illumina) using the Nextera library preparation method with 2 × 150 bp paired end reads. Reads were assembled into contigs using the Shovill pipeline (https://github.com/tseemann/shovill). Sequence types, the presence of resistance genes, and speciation were confirmed, using MLST version 2.0, ResFinder version 4.1 [17], and KmerFinder version 3.2 [18] on the Center for Genomic Epidemiology platform (https://cge.cbs.dtu.dk); contigs were annotated using Prokka [19]. A core genome single-nucleotide polymorphism (SNP) alignment was generated using Parsnp [20] and viewed using Interactive Tree of Life version 6.1.1 [21] using *P. aeruginosa* PAO1 (GenBank accession no. NC_002516) as the reference sequence. SNP distances between the core genomes of all isolates were calculated using snp-dists [22].

Sequence data from this study was submitted to the National Center for Biotechnology Information’s Sequence Read Archive (BioProject no. PRJNA1044010).

## Results and discussion

### Isolate demographics

A total of 44 MBL-producing *P. aeruginosa* isolates were received at the NARA over the 12-month period of the study, and following deduplication (by patient and MBL-type), 39 isolates were retained for further analysis. Half of the isolates were obtained from screening swabs (including faeces), and the remaining isolates were from urine (7/39; 17.9%), wound (7/39; 17.9%), and respiratory (4/39; 10.3%) samples; only a single isolate was obtained from a blood sample. Most isolates were obtained from males (27/39; 69.2%). Isolates were submitted from 11 Swiss Cantons, with the highest numbers of isolates being submitted from Zurich (*n* = 9) and Geneva (*n* = 8), the most populous areas of Switzerland.

### Antibiotic resistance genes and phenotypic analysis

Within the 39 isolates, the VIM-type enzymes were the most frequently identified (*n* = 21) carbapenemases, followed by NDM-type (*n* = 11) and IMP-type (*n* = 6) β-lactamases. Of note, a single isolate produced both a VIM-type and an NDM-type enzyme. All isolates exhibited resistance to ceftazidime (CAZ), imipenem (IPM), meropenem (MEM), and the BLBLIs ceftolozane-tazobactam (TOL-TAZ), ceftazidime-avibactam (CAZ-AVI), imipenem-relebactam (IPM-REL), and meropenem-vaborbactam (MEM-VAB) (Table 1). The lack of activity of these BLBLIs is unsurprising since TAZ, the DBO inhibitors, AVI and REL, and the cyclic boronate inhibitor, VAB, do not exhibit any activity against class B beta-lactamases [23]. Resistance to ATM and the, as yet unlicensed combination, ATM-AVI, was observed in 5 isolates, with MICs of 32 mg/L (*n* = 4) and 256 mg/L. MBLs are known to be unable to hydrolyse the monobactam ATM; however, many class A and C beta-lactamases efficiently hydrolyse this antimicrobial [11]. A total of 19 and 18 isolates showed resistance to the novel -still unlicensed- combinations FEP-TAN and FEP-ZID, using the preliminary breakpoint of 8 mg/L for both. Within the FEP-TAN resistant isolates, six produced IMP-type enzymes, against which TAN is known to exhibit no activity [12]. When combined with the efflux pump inhibitor, PAβN, MIC reductions were observed in all FEP-TAN-R isolates (ranging from 2- to 1024-fold) and in 17/18 FEP-ZID-R isolates (ranging from 2- to 1024-fold). This resulted in 5/19 FEP-TAN-R isolates and 16/18 FEP-ZID-R isolates becoming sensitive. This suggests that resistance to these novel combinations is, at least in part, attributable to efflux mechanisms in *P. aeruginosa*. FDC resistance was observed in 8 isolates, 6 of which produced NDM-type MBLs and two with IMP-type. High-level resistance was observed to ciprofloxacin (36/39; 92.3%) and amikacin (29/39; 74.4%), but all isolates remained susceptible to colistin.

**Table 1:** MIC distributions of MBL-producing *P. aeruginosa* (*n* = 39) from this study

Antimicrobial agent	Breakpoint (mg/L)	Isolates (no./%)											Resistant (no./%)
		≤ 0.25	0.5	1	2	4	8	16	32	64	128	≥ 256	
Ceftazidime	R > 8								**6/15.4**	**5/12.8**	**7/17.9**	**21/53.8**	39/100.0
Ceftazidime-avibactam	R > 8								**6/15.4**	**5/12.8**	**7/17.9**	**21/53.8**	39/100.0
Cefepime	R > 8						7/17.9	**5/12.8**	**3/7.7**	**3/7.7**	**1/2.6**	**20/51.3**	32/82.1
Cefepime-taniborbactam	R > 8			2/5.1	9/23.1	7/17.9	2/5.1	**1/2.6**		**2/5.1**	**3/7.7**	**13/33.3**	19/48.7
Cefepime-zidebactam	R > 8	19/48.7			1/2.6		1/2.6	**3/7.7**	**4/10.3**	**2/5.1**	**4/10.3**	**5/12.8**	18/46.2
Aztreonam	R > 16			1/2.6		7/17.9	5/12.8	21/53.8	**4/10.3**			**1/2.6**	9/23.1
Aztreonam-avibactam	R > 16			1/2.6		8/20.5	7/17.9	18/46.2	**4/10.3**			**1/2.6**	7/17.9
Ceftolozane-tazobactam	R > 4									**3/7.7**	**10/25.6**	**26/66.7**	39/100.0
Imipenem	R > 4								**1/2.6**	**5/12.8**	**2/5.1**	**31/79.5**	39/100.0
Imipenem-relebactam	R > 2								**1/2.6**	**5/12.8**	**2/5.1**	**31/79.5**	39/100.0
Meropenem	R > 8							**3/7.7**	**3/7.7**	**7/17.9**	**5/12.8**	**21/53.8**	39/100.0
Meropenem-vaborbactam	R > 8							**3/7.7**	**4/10.3**	**7/17.9**	**4/10.3**	**21/53.8**	39/100.0
Cefiderocol	R > 2	7/17.9	6/15.4	11/28.2	7/17.9	**7/17.9**		**1/2.6**					8/20.5
Ciprofloxacin	R > 0.5	2/5.1	1/2.6	**1/2.6**		**2/5.1**	**7/17.9**	**6/15.4**	**6/15.4**	**8/20.5**	**3/7.7**	**3/7.7**	36/92.3
Amikacin	R > 16			2/5.1	1/2.6	1/2.6	2/5.1	4/10.3	**6/15.4**	**2/5.1**	**6/15.4**	**15/38.5**	29/74.4
Colistin	R > 4	36/92.3	3/7.7										0/0.0

Bold values illustrate resistant MICs

### FDC resistance and MBL production

To assess the contribution of MBLs to FDC non-susceptibility, MICs were performed on recombinant *P. aeruginosa* strains producing *bla*_NDM-1_, *bla*_IMP-1_, and *bla*_VIM-2_. Strains producing NDM-1 and IMP-1 were resistant with MICs respectively at 16 and 8 mg/L, while the VIM-2-producing recombinant strain exhibited an MIC at 1 mg/L (Table 2), corresponding to a notable increase (4-fold), despite remaining sensitive. These results suggest that the MBL types produced in the 8 FDC-resistant strains in this study contribute significantly to this phenotype.

**Table 2:** FDC MICs of recombinant *P. aeruginosa* strains producing the MBLs, NDM-1, IMP-1, and VIM-2

Strain	FDC MIC (mg/L)
PA14	0.25
PA14/pUCP24	0.25
PA14/pUCP-NDM-1	16
PA14/pUCP-IMP-1	8
PA14/pUCP-VIM-2	1

### Whole-genome sequence analysis

#### STs and carbapenemase genes

WGS of 34 isolates identified the following MBLs: *bla*_NDM-1_ (*n* = 11), *bla*_VIM-2_ (*n* = 8), *bla*_IMP-1_ (*n* = 4), *bla*_VIM-4_ (*n* = 4), *bla*_VIM-5_ (*n* = 2), and *bla*_VIM-36_ (*n* = 2), and individual isolates each carried *bla*_IMP-7_, *bla*_IMP-13_, and *bla*_NDM-1_ + *bla*_VIM-2_. Thirteen different STs were found with ST773 being the most prevalent with all eight isolates producing *bla*_NDM-1_ and submitted from 5 cantons. Seven ST111 isolates were identified, harbouring either *bla*_VIM-2_ (*n* = 4) or *bla*_VIM-4_ (*n* = 3) respectively, and four ST1047 isolates harboured *bla*_IMP-1_. All other STs were represented by ≤ 2 isolates. Among the 13 STs identified, sixteen isolates represented by six STs (STs 111, 235, 298, 308, 357, and 654), are members of the worldwide top 10 high-risk clones [3]. These findings are similar to those found in a UK study, analysing PA-MBL isolates collected from 2003 to 2012, where STs 111, 235, 233, 357, 654, and 773 were found to be dominant, and VIM-type enzymes were the major MBL-type [24]. These similarities between both studies, despite the difference in the collection periods, illustrate the long-term global dominance and stability of these *P. aeruginosa* high-risk clone lineages. Among the 8 isolates that exhibited resistance to FDC, 6 produced NDM-1 (of which 5 were ST773), a single isolate produced IMP-1, and another produced IMP-7. Production of NDM enzymes has previously been associated with elevated FDC MICs in *P. aeruginosa* and Enterobacterales [9, 10]. Taken alongside the data obtained from the expression of *bla*_IMP-1_ in a recombinant *P. aeruginosa* strain above, it could therefore be concluded that IMP-type enzymes likely contribute to decreased susceptibility to FDC in these isolates.

**Fig. 1:**
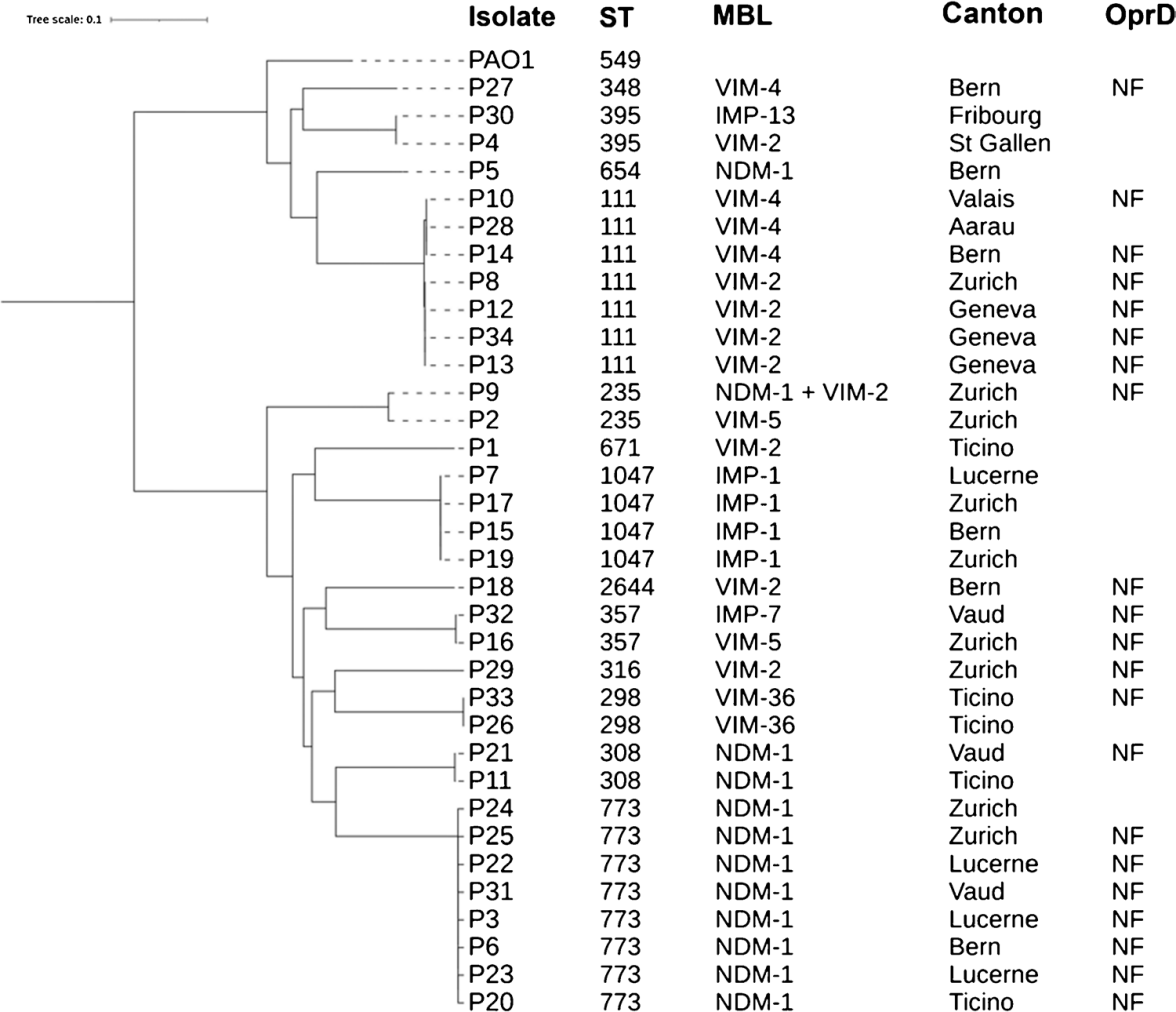
Core genome alignment of 34 MBL-producing *P. aeruginosa* strains with STs, MBL variants, Canton of origin and OprF status. NF; non-functional, blank; functional

A core genome alignment of all 34 sequenced isolates (Fig. 1) showed the clustering of isolates sharing the same ST and MBL but from different cantons. However, analysis of the SNP distances between the clusters of isolates sharing the same ST and MBL did not evidence any obvious epidemiological link, with differences ranging from 196 to 1343 SNPs. This illustrates that despite the commonalities (STs and MBLs) between these clusters of isolates, none were as a result of a clonal outbreak, and instead this highlights the dominance of these particular STs in MBL-PA.

#### OprD and PBPs

Analysis of the *oprD* gene sequences identified that most isolates produced a non-functional OprD, most often due to mutations resulting in truncated proteins. The OprD porin is a well-known route by which imipenem can enter the *P. aeruginosa* cell [4], and this was evidenced by all isolates with non-functional OprD exhibiting imipenem MICs ≥ 128 mg/L (128->256 mg/L), while those with a functional OprD showed lower average MICs (32–256 mg/L). Analysis of PBP-2 and PBP-3, known beta-lactam targets and particularly associated with resistance to some of the BLBLIs, did not reveal any mutations.

#### RMTases

RMTase-encoding genes were detected in 10 isolates, among which nine isolates harboured *rmtB,* including eight isolates which produced NDM-1 (all ST773), and a single isolate that produced VIM-2 (ST316). In addition, one isolate, an ST308 NDM-1 producing strain, also produced the RmtD RMTase. The high-risk clone ST773 had previously been reported to carry *rmtB* and *bla*_NDM-1_ in isolates from the UK [25]. RMTases can confer high level resistance to all clinically relevant aminoglycoside antibiotics, and therefore monitoring of their prevalence is essential.

## Conclusions

MBL-PA are increasing in incidence globally, including in Switzerland. The isolates in this study were frequently found to be resistant to most beta-lactams, as well as non-beta-lactam antibiotics including fluoroquinolones and aminoglycosides. The levels of resistance observed to the novel BLBLI combinations (ATM-AVI, FEP-TAN, FEP-ZID), not currently in use and still under development, are concerning and illustrate the challenges faced when treating infections caused by MBL-PA. This study showed the wide dissemination in Switzerland of dominant *P. aeruginosa* high-risk clones that accounted for the majority of isolates in this study. The co-production of 16S rRNA methylases in many of these MBL producers is a significant concern, since these two mechanisms contribute to the pan-resistance to almost all beta-lactams and aminoglycosides, both antibiotic families being critical for the treatment of the corresponding infections.

### Supplementary information

ESM 1 (DOCX 12 kb)

## Data Availability

Sequence data from this study was submitted to the National Center for Biotechnology Information’s Sequence Read Archive (BioProject no. PRJNA1044010).

## References

[1] Tenover FC, Nicolau DP, Gill CM (2022). Carbapenemase-producing Pseudomonas aeruginosa - an emerging challenge. Emerg Microbes Infect.

[2] Juan C, Peña C, Oliver A (2017). Host and Pathogen biomarkers for severe Pseudomonas aeruginosa infections. J Infect Dis.

[3] Del Barrio-Tofiño E, López-Causapé C, Oliver A (2020). Pseudomonas aeruginosa epidemic high-risk clones and their association with horizontally-acquired β-lactamases: 2020 update. Int J Antimicrob Agents.

[4] Li H, Luo YF, Williams BJ, Blackwell TS, Xie CM (2012). Structure and function of OprD protein in Pseudomonas aeruginosa: from antibiotic resistance to novel therapies. Int J Med Microbiol.

[5] Horcajada JP, Montero M, Oliver A, Sorlí L, Luque S, Gómez-Zorrilla S, Benito N, Grau S (2019). Epidemiology and treatment of multidrug-resistant and extensively drug-resistant Pseudomonas aeruginosa infections. Clin Microbiol Rev.

[6] Treepong P, Kos VN, Guyeux C, Blanc DS, Bertrand X, Valot B, Hocquet D (2018). Global emergence of the widespread Pseudomonas aeruginosa ST235 clone. Clin Microbiol Infect.

[7] Canton R, Doi Y, Simner PJ (2022). Treatment of carbapenem-resistant Pseudomonas aeruginosa infections: a case for cefiderocol. Expert Rev Anti-Infect Ther.

[8] Jorth P, McLean K, Ratjen A, Secor PR, Bautista GE, Ravishankar S, Rezayat A, Garudathri J, Harrison JJ, Harwood RA, Penewit K, Waalkes A, Singh PK, Salipante SJ (2017). Evolved aztreonam resistance is multifactorial and can produce hypervirulence in Pseudomonas aeruginosa. mBio..

[9] Poirel L, de la Rosa JM O, Sadek M, Nordmann P (2022). Impact of acquired broad-spectrum β-lactamases on susceptibility to cefiderocol and newly developed β-lactam/β-lactamase inhibitor combinations in Escherichia coli and Pseudomonas aeruginosa. Antimicrob Agents Chemother.

[10] Fröhlich C, Sørum V, Tokuriki N, Johnsen PJ, Samuelsen Ø (2022). Evolution of β-lactamase-mediated cefiderocol resistance. J Antimicrob Chemother.

[11] Karlowsky JA, Kazmierczak KM, de Jonge BLM, Hackel MA, Sahm DF, Bradford PA (2017). In vitro activity of aztreonam-avibactam against Enterobacteriaceae and Pseudomonas aeruginosa isolated by clinical laboratories in 40 countries from 2012 to 2015. Antimicrob Agents Chemother.

[12] Liu B, Trout REL, Chu GH, McGarry D, Jackson RW, Hamrick JC, Daigle DM, Cusick SM, Pozzi C, De Luca F, Benvenuti M, Mangani S, Docquier JD, Weiss WJ, Pevear DC, Xerri L, Burns CJ (2020). Discovery of taniborbactam (VNRX-5133): a broad-spectrum serine- and metallo-β-lactamase inhibitor for carbapenem-resistant bacterial infections. J Med Chem.

[13] Sader HS, Mendes RE, Duncan LR, Carvalhaes CG, Castanheria M (2022). Antimicrobial activity of cefepime/zidebactam (WCK 5222), a β-lactam/β-lactam enhancer combination, against clinical isolates of Gram-negative bacteria collected worldwide (2018-19). J Antimicrob Chemother.

[14] EUCAST. Clinical breakpoint table v.13.1. 2023. https://www.eucast.org/fileadmin/src/media/PDFs/EUCAST_files/Breakpoint_tables/v_13.1_Breakpoint_Tables.pdf. Accessed 9 Nov 2023

[15] Dortet L, Poirel L, Nordmann P (2012). Rapid detection of carbapenemase-producing Pseudomonas spp. J Clin Microbiol.

[16] West SE, Schweizer HP, Dall C, Sample AK, Runyen-Janecky LJ (1994). Construction of improved Escherichia-Pseudomonas shuttle vectors derived from pUC18/19 and sequence of the region required for their replication in Pseudomonas aeruginosa. Gene..

[17] Zankari E, Hasman H, Cosentino S, Vestergaard M, Rasmussen S, Lund O (2012). Identification of acquired antimicrobial resistance genes. J Antimicrob Chemother.

[18] Larsen MV, Cosentino S, Lukjancenko O, Saputra D, Rasmussen S, Hasman H (2014). Benchmarking of methods for genomic taxonomy. J Clin Microbiol.

[19] Seeman T (2014). Prokka: rapid prokaryotic genome annotation. Bioinf..

[20] Treangen TJ, Ondov BD, Koren S, Phillippy AM (2014). The Harvest suite for rapid core-genome alignment and visualization of thousands of intraspecific microbial genomes. Genome Biol.

[21] Letunic I, Bork P (2019). Interactive Tree Of Life (iTOL) v4: recent updates and new developments. Nucleic Acids Res.

[22] Seemann T (2019) snp-dists. In: GitHub repository. GitHub https://github.com/tseemann/snp-dists. Accessed 9 Nov 2023

[23] Papp-Wallace KM (2019). The latest advances in β-lactam/β-lactamase inhibitor combinations for the treatment of Gram-negative bacterial infections. Expert Opin Pharmacother.

[24] Wright LL, Turton JF, Livermore DM, Hopkins KL, Woodford N (2015). Dominance of international 'high-risk clones' among metallo-β-lactamase-producing Pseudomonas aeruginosa in the UK. J Antimicrob Chemother.

[25] Taylor E, Bal AM, Balakrishnan I, Brown NM, Burns P, Clark M, Diggle M, Donaldson H, Eltringham I, Folb J, Gadsby N, Macleod M, Ratnaraja NVDV, Williams C, Wootton M, Sriskandan S, Woodford N, Hopkins KL (2021). A prospective surveillance study to determine the prevalence of 16S rRNA methyltransferase-producing Gram-negative bacteria in the UK. J Antimicrob Chemother.

