# Supplementary material for: Molecular analysis of metallo-beta-lactamase-producing Pseudomonas aeruginosa in Switzerland 2022–2023
Source: Eur J Clin Microbiol Infect Dis. 2024 Jan 18;43(3):551–7. doi: 10.1007/s10096-024-04752-8 (PMC10917820; doi:10.1007/s10096-024-04752-8)
Supplement: Supplementary file 1 — (DOCX 12 kb) [file 10096_2024_4752_MOESM1_ESM.docx]

| **Gene Target** | **Forward Primer** | **Reverse Primer** |
| --- | --- | --- |
| *bla*_NDM-1_ | GATGATGAGCTCGGAAAACTTGATGGAATTGC | GATGATGGATCCACGTGGTCAGCCATGGCTCA |
| *bla*_IMP-1_ | GACGACGAGCTCTACCGTAACCACCCCAGATG | GACGACCTGCAGCAAACTGTCCAGCCACGTAG |
| *bla*_VIM-2_ | GATGATGAGCTCGTTATGGAGCAGCAACGATG | GATGATGGATCCGCAACTTCATGTTATGCCGC |

**Table S1.** Primers used for amplification of MBLs for cloning into pUCP24.
